# Involving Citizens in Integrated Care. Lessons Learned Through Participatory Action Research in Three Communities in the Netherlands

**DOI:** 10.5334/ijic.9026

**Published:** 2026-04-16

**Authors:** Geert M. Rutten, Ellen van Wijk, Saskia Sleijster, Dorien L. Oostra, Janine Roenhorst, Marloes Kleinjan, Miranda Laurant

**Affiliations:** 1 School of Health Studies, HAN University of Applied Science, P.O. Box 6960, NL 6503GL Nijmegen, The Netherlands; 2 Miranda Laurant Coaching and Healing, Druten, The Netherlands

**Keywords:** citizen involvement, integrated care, cross-domain collaboration

## Abstract

**Introduction:**

In response to rising healthcare demands, alternative health system models are needed. This study aims to investigate the development of community-involved, cross-domain networks, and to identify factors that facilitate or hamper their growth and sustainability.

**Methods:**

This participatory action research used purposive sampling to select three diverse networks. A multi-methods approach of document analysis, observations, dialogue and twenty semi-structured interviews across the networks was applied. The ESSA-framework (Exploring, Shaping, Strengthening, Anchoring) of network development was used to track progression across its stages. Reflexivity was ensured through discussions with the networks, an advisory board, and the study’s steering committee.

**Results:**

All networks advanced by one ESSA phase; however, one only progressed in interprofessional collaboration. Key factors affecting network development included a shared sense of urgency and community, mode of collaboration, essential roles, and a mix of competencies.

**Discussion:**

The limited number of networks limits external validity, yet their diversity, and the mixed methods and reflexivity revealed profound insights into community-involved cross-domain network development.

**Conclusion:**

Community-involved cross-domain network development is complex and requires felt urgency, patience and perseverance from all participants. Early engagement of both professionals and citizens is required, supported by network leadership and change-agent skills to drive development.

## Introduction

In many countries, the demand for healthcare is increasing. In high income countries, this trend is largely driven by an ageing population, the rising prevalence of chronic conditions and multimorbidity, and higher complexity of health problems [1,2]. It has also been established that non-medical issues, such as loneliness, unemployment or financial problems, contribute substantially to the complex health problems seen by general practitioners (GP’s) [3,4]. Indeed, research indicates that as much as 90 % of what people need to feel healthy lies outside the medical domain, and is instead rooted in psychological and social dimensions [5].

The urgency of these challenges is reflected in the Netherlands, where the Scientific Council for Government Policy concluded in 2021 that the sustainability of the healthcare system is under pressure due to high costs, declining public support, and persistent workforce shortages [6]. This trend is not unique to the Netherlands: globally, a substantial lack of workforce is expected in 2030 [7,8]. In the Netherlands alone, about 59.000 healthcare and welfare vacancies were unfulfilled in 2024 [9].

In response, policymakers and health and welfare professionals have sought solutions that keep care and support accessible and appropriate. Strategies include providing care closer to people’s living environment and strengthening people’s ability to manage their own health [10]. However, shifting hospital care into community settings has proven difficult [11,12,13], and may place additional burdens on already overstretched general practitioners (GPs) [14].

In their Conceptual Framework for Integrated Community Care (ICC), Thiam et al. emphasize the importance of an integration of health and social care and identified the role of the spatial and temporal context, the spatial and relational proximity in care delivery, and the fact that care should be customized to the needs of the inhabitants of a region [15]. Community engagement is not only ethically relevant, but also effective: research shows that layperson-led initiatives and community involvement can improve health outcomes, particularly among disadvantaged groups [16,17]. Community health workers, who act as bridges between residents, citizen initiatives, and healthcare professionals, are promising actors in this regard and may help reduce socioeconomic health inequalities [18,19].

Nevertheless, evidence on the effectiveness of integrated care networks remains mixed. While some studies suggest that such networks can reduce healthcare utilization [20,21], systematic reviews conclude that the overall evidence base is still inconclusive [22]. A key knowledge gap concerns *how* community-involved, cross-domain networks, i.e. collaborative networks in which community members and professionals from multiple sectors — such as healthcare and social services — work together, actually emerge and develop over time, and which factors facilitate or hinder their sustainability.

To address this gap, this study applies the ESSA-model of network formation [23]. The ESSA-model describes four iterative stages—Exploring, Shaping, Strengthening, and Anchoring—that help explain how collaborative, cross-domain networks evolve. Facilitating meaningful community involvement in these networks requires effective communication, a proactive approach, peer facilitators to reach underserved groups, and support from organizations and local leaders. It also demands sensitivity to culture and language, shared decision-making, and recognition that building trust takes time and effort of all parties involved [24,25]. A phased and transparent process from the outset is therefore recommended, ensuring that community needs and expectations are acknowledged while trust and collaboration are gradually established [24].

GPs are often the first point of contact for individuals with complex, partly non-medical problems, but they cannot address these issues alone. By applying the ICC-framework and the ESSA-model we can better understand how citizens and professionals such as GPs, social workers and community support workers interact in the development of community-involved cross-domain care and support networks. Community support workers (CSWs) are community members with strong social brokering qualities, that may play a complementary role by linking residents to informal resources, citizen initiatives, and social and public services that extend beyond the medical domain. Community-involved cross domain networks provide the structural backbone that connects these actors, ensuring that GPs are supported rather than overburdened and that citizens’ own capacities and initiatives are meaningfully included. Understanding what is necessary to build sustainable, community-involved cross domain networks that bridge formal and informal care is crucial. Hence, this study aims to investigate the development of community-involved, cross-domain care and support networks in practice, and to identify factors that facilitate or hamper their growth and sustainability.

## Methods

### Design and recruitment of the networks

This study represents a participatory action research (PAR) approach. PAR integrates knowledge generation and practice development in an interconnected process, aiming to enhance the empowerment of participants to address problems that are relevant to the community. As a consequence, it includes research objectives and practice development objectives [26]. PAR emphasizes involving all relevant parties in all phases of the scientific process and includes the action researcher as a facilitator operating closely with network members [27]. Therefore, healthcare and welfare professionals and community members were involved in the execution, data interpretation, and reporting of the study.

**Figure 1:**
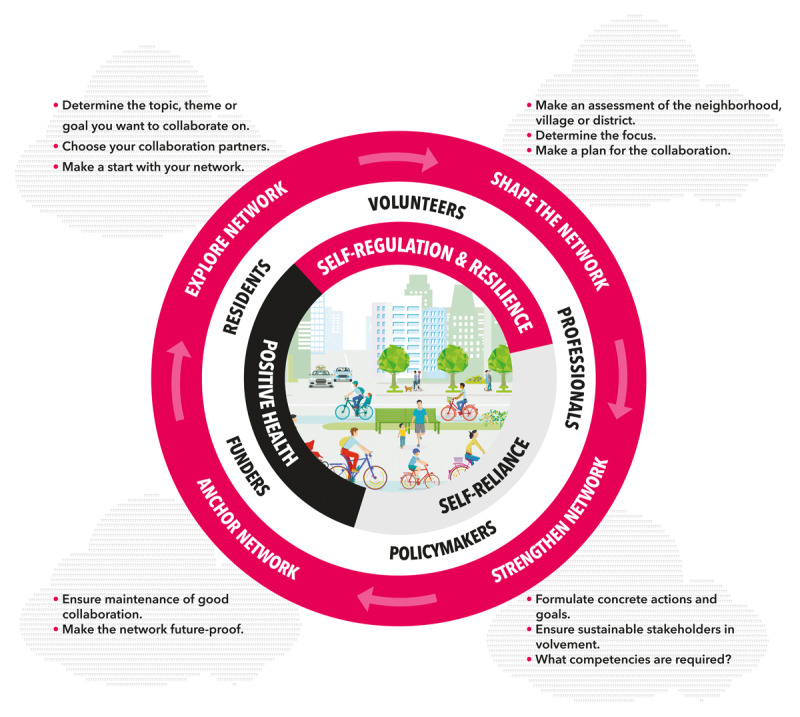
ESSA-model of network formation.

Cross-domain networks were identified through the researchers’ professional connections, the member organisations of the Foundation for Development of Quality Care in General Practice, and the members of the study’s steering committee. A purposive sampling strategy – appropriate for qualitative research with information-rich cases, was applied to ensure diversity across networks [28,29]. The selection aimed to capture network variation in 1) context: rural and urban settings, 2) member characteristics: socioeconomic statuses and ethnic diversity, and 3) status: stage of network formation according to the ESSA model: Exploring, Shaping, Strengthening, and Anchoring (Figure 1 [23]).

Representatives of seven networks were approached to outline research objectives, participation expectations (e.g., time commitment for meetings and data collection), and potential advantages (e.g., support in network development) and disadvantages (e.g., time burden) of participation. If deemed relevant network representatives consulted their network members before confirming participation. Three networks consented to participate; four declined due to competing priorities or time constraints. Enrolment of network members who participated in the study was confirmed upon receipt of informed consent.

### Characteristics of the included networks

The included networks provided sufficient variation across the purposive sampling criteria and were situated in the central and southern part of the Netherlands (Table 1).

**Table 1:** Characteristics of the included networks.


	RURAL VILLAGE (A)	URBAN DISTRICT (B)	SMALL MUNICIPALITY (C)

**Context**	agricultural; socially active community, strong social cohesion	city; various health related and social challenges; high demand for healthcare	agricultural; GP seeks to prevent (future) challenges in the provision of care

**Population**	homogeneous	multicultural > 60 nationalities	low diversity

**Number of residents**	2.100	16.000	21.000

**Income level** ^#^	low to average	very low to low	average

**Network**	care cooperative: Vitality cooperative	interprofessional healthcare network	health cooperative

**Network initiator**	high to medium educated group of citizens	primary care health centre	primary care physician with small group of high educated citizens

**Network stage** ^##^	strengthening	shaping	exploring


^#^ Income level compared to nationwide income level (average income in the Netherlands in 2025 is just over €46,000 gross per year. Source: Netherlands Bureau for Economic Policy Analysis (CPB)). ^##^ Related to ESSA-Model.

The three networks defined the following development objectives:

- Rural village (network A): The Vitality cooperative wants to provide community care and become a recognized partner in healthcare and welfare support with control retained by the Vitality cooperative.
- Urban district (network B): Improve collaboration of healthcare professionals working at the health centre with welfare professionals and the citizens in the community; reduce the burden on healthcare professionals.
- Small municipality (network C): Involve citizens and enhance the collaboration between citizens and primary healthcare professionals to promote health and vitality within the community.

### Theoretical framework

To evaluate the development of the three networks the ESSA-model of network formation was applied (Figure 1 [23]). Its four non-linear, iterative stages of network formation are characterized by different features and corresponding activities.

In the *exploring stage*, initiating partners establish relationships, define the collaboration’s purpose and structure, and identify relevant stakeholders. Key activities include formulating a mission, vision and setting priorities.

The *shaping stage* focuses on analysing the local neighbourhood or village and refining the mission and vision into a plan. Important activities include identifying a driving force and liaison for the network and considering sustainability.

In the *strengthening stage* goals and actions are specified, and collaborative relationships are consolidated. Network expansion often occurs in this stage, increasing the networks capacity to achieve goals. Connecting with citizen initiatives, choosing concrete actions and keeping partners engaged are key activities.

The *anchoring stage* emphasizes sustainability by activities such as reinforcing collaborative foundations, monitoring progress, and securing resources for continuity.

The ESSA model thus provides a structured framework that supports networks in identifying next steps and strategies to strengthen collaboration and enables analysis of their evolution over time.

### Data collection

Between September 2022 and December 2023, three researchers (DO, EvW, SS) were individually assigned to one of the three networks. Consistent with PAR, they simultaneously supported the development of these networks and collected data. To support network development the action researchers suggested and facilitated – in co-creation with the network members – the application of different strategies, based on the actual needs observed or expressed by the network members during the researchers’ biweekly site visits. For data collection multiple qualitative methods were employed, including observations and dialogues during biweekly site visits, document analysis, and semi-structured individual and group interviews conducted at various times during the course of the study. Observations and dialogues focused mainly on network development processes, roles within the networks, stakeholder engagement and factors influencing network development. Analyzed documents comprised mission and vision statements and plans. Data from these sources were compiled in logbooks. The multi-method approach of data collection facilitated data triangulation.

Interviews were informed by an interview guide inspired by scientific literature on Integrated Care, such as the ICC-framework [15], the Rainbow Model of Integrated Care [30] and community health development [31]. The interview guide was discussed with experts in the field of integrated care and community development and considered comprehensive. Topics included enabling conditions of interprofessional and cross-domain collaboration, engagement of citizen initiatives and perceived outcomes of cross-domain collaboration. The interviews lasted approximately one hour. Written informed consent was obtained from all participants.

### Participants in the interviews

Between July 2023 and December 2023, twenty interviews were held in a convenience sample of twenty-nine participants involved in the networks (Table 2). Eleven participants were citizens of whom three were also community support workers. Four were general practitioners, one nurse practitioner, five GP-assistants and two community nurses. Also, one change agent, two project leaders and two healthcare managers participated.

**Table 2:** Participants in the interviews.


NETWORK	TYPE OF INTERVIEW	NUMBER OF INTERVIEWS	PARTICIPANTS

Rural community (A)	Duo interview	1	General Practitioner Nurse practitioner

	Group interview	1	Community support workers (citizen; n = 2) Community nurse Former community support worker (citizen)

	Individual interviews	2	External advisor citizen initiative (change agent) Community nurse

Urban district (B)	Group interview	1	GP assistant Community support worker (citizen) Manager homecare Manager general practitioner

	Individual interviews	9	GP assistants (n = 4) General practitioner (n = 2) Citizens (n = 3)

	Duo interview	1	Project leaders (n = 2)

Small Municipality (C)	Individual interviews	4	General Practitioner Citizen (n = 2 of whom one is also care and welfare advisor) Project leader

	Duo interview	1	Citizens (n = 2)


### Data analysis

Interviews were audio-recorded, transcribed verbatim and thematically analysed using an inductive approach [32]. This allowed for the identification of concepts and the exploration of the complex interrelationships and processes inherent in the development of cross-domain networks [33]. Data-analysis was conducted with four researchers (EvW, DO, SS, JR), who coded the interviews independently. Initial open coding was followed by axial and selective coding in several rounds. After each round findings were discussed in a meeting with participation of the project supervisors (GR, ML) to reach consensus, refine the codes and identify key concepts. Interviews were analyzed using Atlas.ti version 23 for qualitative data analysis [34].

Logbook data were analyzed using qualitative content analysis with a directed approach to supplement and enrich the interview results [35]. The stages of the ESSA-model and the results of the interview analysis were used as a lead, but the approach allows for the identification of new themes.

Integrating the findings from the various data sources resulted in an initial set of concepts for community-involved cross-domain care and support networks, which could be presented for discussion to the network members, the advisory board, and the steering committee.

### Reflexivity

The six researchers had diverse backgrounds in nursing, allied healthcare, social work and healthcare management. To prevent one-sided interpretation, findings were regularly presented to and discussed with various project stakeholders ensuring broadly supported results. During every research phase, i.e. preparation, action and data collection, reflection, data analysis and reporting, an advisory board composed of ten members with a professional background in network formation facilitation, primary healthcare, social work, policy and three citizens was involved. Citizens were important partners because they could contribute their experiential knowledge as community members and care recipients. During the study member-check meetings with the advisory board were held approximately every six months to discuss, interpret, and reflect on findings and conclusions before finalizing the results. Intermediate and final results were also discussed during twelve meetings with the study’s steering committee, comprising experts in healthcare, welfare, local and national policy, citizen initiatives, and higher professional education. Members of each involved network and the advisory board participated in a learning community initiated by the researchers, enabling the networks to learn from each other. The final learning community meeting also served as a platform for identification and establishment of overarching themes. Throughout the research process, power dynamics inherent in PAR were addressed through regular reflection on researcher positionality. Also, special attention was paid to the equal contribution of participants during meetings.

The study was approved by the Research Ethics Committee of the HAN University of Applied Sciences (file nr. ECO 306.11/21).

## Results

### Development of the networks

Logbook analysis revealed that after fifteen months of participation, the three networks progressed in their development. Strategies applied to support network development were tailored to the needs that emerged or were expressed by the network members during the researchers’ biweekly site visits (Table 3).

**Table 3:** Strategies applied to support development of the three networks.


STRATEGIES	RURAL VILLAGE (A)	URBAN DISTRICT (B)	SMALL MUNICIPALITY (C)

Analysis of potential partners for collaboration	✓		

Organizing the dialogue with partners to reach mutual understanding	✓		

Co-creating a tool to measure the value of the interventions	✓		

Supporting the organization of meetings with healthcare professionals and/or social workers and/or citizens	✓		✓

The establishment and organisation of dialogue meetings with the sounding board group of citizens		✓	

A stakeholder- and force-field analysis, stakeholder interviews		✓	✓

Strength and Weakness (SWOT)-analysis	✓	✓	

Feedback on documents (e.g. job descriptions; a community care plan)	✓	✓	

Supporting the development of new strategic network objectives		✓	

Dialogue sessions with the initiators of the network to share research findings	✓	✓	✓


The Vitality cooperative in the small rural village (network A) was now shifting from the strengthening towards the anchoring stage and made important steps towards their development objective of becoming a recognized partner in healthcare and social support. They established their own care team including a community nurse, two community support workers and two home care professionals. The cooperation also established to be recognized as a formal partner in delivering care and support under the Social Support Act. Finally, they developed criteria for collaboration with their preferred home care organization. Collaboration with the GP was initiated but was still a point of attention.

The health centre in the multicultural low SES urban district (network B) moved to the strengthening stage with regard to their collaboration with healthcare and welfare professionals. The network also changed towards a more planned instead of an intuitive approach in their network development process. However, collaboration with citizens moved backward to the exploration stage. A previously installed sounding board group including four Dutch, one Turkish and one Somali resident was cancelled due to GPs’ time constraints. With regard to their development objective, which sought to enhance collaboration with both health and welfare professionals as well as citizens, the network had thus achieved only partial progress. However, collaboration with citizens was still an important objective of the health centre. Therefore, they planned to improve their collaboration with a welfare organization as a bridge to citizens.

In the small municipality (network C) the care cooperative evolved from the exploration to the shaping stage. Initial collaborations with citizens and professionals indicate that the cooperative is progressing towards its development objective, though the contribution to a healthier community can only be determined in the long term. The network’s initial top-down approach and need for control, delayed recognition of the researcher as a legitimate contributor and hampered citizen involvement. Following discussion with the researcher the initiators adopted a more community-up strategy. They engaged citizens to better align with the community’s needs and opportunities and fostered early connection between citizens and healthcare and welfare professionals. This resulted in the establishment of voluntary neighbourhood liaisons, supervised by a social worker and the GP, to strengthen social cohesion in the neighbourhoods. To enhance visibility, the care cooperative launched an online platform for neighbourhood participation, organised information meetings on relevant health topics and intensified their collaboration with a local welfare organisation.

Across the three networks, we observed that professional led networks (top-down; network B and C) often prioritized building their professional networks before engaging with citizen initiatives. Conversely, the citizen led network (bottom-up; network A) strengthened their community network before reaching out to professionals. In both approaches it proved difficult to establish a connection between formal and informal care and support.

### Factors influencing cross-domain network development

**Figure 2:**
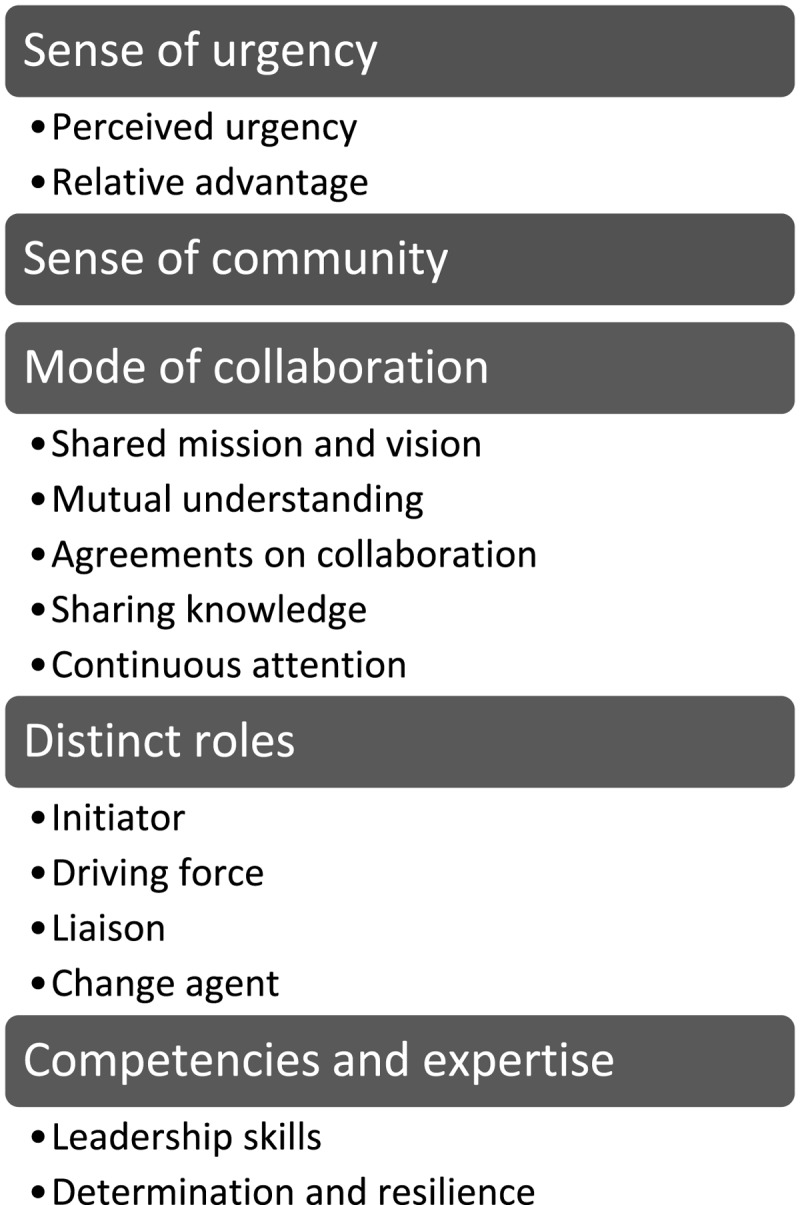
Factors influencing collaboration.

Related to the research objective of the study, the PAR within the three networks, along with the input from the advisory board, the steering committee and the learning community yielded five overarching themes, each including several factors influencing collaboration in community involved cross domain networks (Figure 2).

### Sense of urgency

During the interviews, various professionals and citizens mentioned that professionals and residents must either perceive a *sense of urgency* regarding the topic or recognize a clear *added value* of the collaboration compared to the current situation. With the exception of the rural village (network A), which was initiated by citizens, it was noticed that citizens often did not share the top-down sense of urgency for change, which impeded their ability to experience the added value of collaboration. Logbook data revealed that, for instance, in network C only after reaching out to the citizens, and identifying their needs, they became active participants in the network.

*‘[…] especially where we are heading in the future, with those significant shortages (in workforce) and the number of requests coming our way. We will need to work together to empower people to take control of their own lives and to make use of informal care.’ (manager homecare, network B)*

*‘You would need to find some kind of shared interest, and I think that’s very difficult. However, in a street or a neighborhood, there are sometimes shared interests, frustrations, or desires.’ (citizen, network C)*

### Sense of community

Mainly citizens and community support workers highlighted a *sense of community*, i.e. the feeling of being a member of a social system, during interviews and dialogues as another important factor for successful citizens’ engagement and their willingness to help each other. Logbook data showed that in contrast to the urban district and the small municipality, the rural village already demonstrated a strong sense of community which was reflected in the citizens’ willingness to take care of each other.

*‘It will only work if people feel it and are willing to do it for each other.’ (GP, Network C)*

*‘This will only work when people in the neighborhood feel strongly connected.’ (citizen, Network A)*

### Mode of collaboration

*Shared Mission and Vision*: During the interviews, members of all three networks revealed that a clear, shared mission and vision with concrete objectives contributes to network formation, enhancing networks’ ability to achieve desired outcomes. Communicating core values, mission, vision, and objectives motivates partners to participate and helps in decision-making and justification. This was supported by the observation that the mission and vision statement of the Vitality cooperation in the rural village served as their compass by which they assessed the suitability of new activities for their purpose.

*‘We have been working on this for about 10 years now. Sometimes we succeed, and sometimes we don’t. […] Our mission and vision document serves as our guide. It helps us stay on track and avoid getting sidetracked.’ (community support worker, Network A)*

The network partners in the urban district noticed that developing a shared mission and vision also supports a sense of ownership among the partners, which is important for the formation of a sustainable network.

*‘[…] and that’s really important to ensure there’s no duplication, […] to share the same vision so that the resident is approached in a consistent way. It’s about understanding what’s being asked, what we can offer, and I think that’s essential in the whole process.’ (manager homecare, Network B)*

*Mutual understanding*: Network partners in all three networks noticed that partners from different domains use different languages depending on their professional or citizen background. For example, observations during joint meetings showed that healthcare professionals, social workers and citizens attributed different meanings to terms such as prevention and community. Therefore, to reduce the risk of miscommunication and misunderstandings, which impedes the development of a sound collaboration, they realized that they had to invest in mutual understanding. Improving mutual understanding also helped them to build *trust*.

*‘Language is essential when discussing core concepts: constantly ask yourself what you and others mean by the terms being used. Some concepts that we consider perfectly normal in healthcare may not align with the perspective of citizens or social workers.’ (GP, network C)*

*Agreements on collaboration*: Networks also need to make agreements about their collaboration. For sustainability, agreements on *equal partnership and reciprocity* are supportive. During an initial meeting with citizens and professionals in the small municipality, it was observed that the professionals’ top-down approach led citizens to lose interest in participation. In a subsequent meeting, citizens were asked what they considered important, which resulted in greater enthusiasm and a more active exchange of ideas.

*‘At first, it felt like professionals were adding their problems to our plate, but that is not how it works. It’s important that you feel intrinsically motivated to want this—not just to solve someone else’s problems. Otherwise, it feels like an imposed initiative, like ‘you have to organize this yourselves’. (citizen, network C)*

Because the energy is in the action, the three networks repeatedly expressed during meetings and dialogues that they were keen to avoid becoming primarily a discussion group. They indicated that avoiding endless discussions and striving for quick wins would maintain participants’ energy and motivation.

*‘I didn’t come here to join a discussion group; I want to take action, roll up my sleeves, and get things done.’ (citizen, network A)*

*Sharing knowledge*: The activities in the networks emphasized the importance of mutual learning and knowledge sharing to accelerate the development of sustainable collaboration in neighborhood networks. Also, some professionals and citizens indicated in the interviews that policy makers need to *facilitate the change process*, for instance by informing the public about the challenges that health care is facing, making existing knowledge available on a central platform and *provide the opportunity to experiment* with new solutions by slightly stretching the strict operational boundaries of the different domains.

*‘I enjoyed the organized meetings with the other networks in this project. Hearing from them about how they approached things and dealt with setbacks was really valuable. And it’s also nice to be able to share your own successes.’ (community support worker, network A)*

*Continuous attention*: Interviews and logbooks revealed that sustaining collaboration in networks is a process that never ends and requires continuous attention and investments. Due to the voluntary character of citizen participation, citizen involved networks are not stable in composition and dynamics. For example, the community support worker in the rural village retired and was replaced by two new community support workers who had to find their way in the community. Also, network objectives evolve over time, changing the importance of stakeholders or requiring new stakeholders in the network. For instance, because of the plan to establish a connection with citizens through social workers in the urban district, the welfare organization became an important stakeholder and network partner.

*‘We are planning to offer home care services through our community cooperative soon. Therefore, we are currently in discussions with a home care organization with whom we want to collaborate, so they can serve as our backup when needed.’ (community support worker, Network A)*

### Distinct roles

The logbooks showed that thorough evaluation of stakeholders and competencies is essential to create value in the network. Because objectives evolve over time, such analysis must be repeated at various stages of network development and beyond to onboard the right partners to achieve the objectives. Several key roles in establishing and maintaining cross-domain collaboration were identified.

*Initiator:* Logbooks and interviews indicated the importance of an initiator in starting the network and inspiring others to commit. Logbooks further showed that the initiator can be either an individual or a small group. In the small municipality the initiator was a general practitioner, but in the other two networks it was a small group of individuals who shared a common vision for establishing a cross-domain network.

*Driving force*: Several interviewees mentioned the importance of a driving force to keep the process going and participants engaged. Logbooks showed that in the rural village the community support worker and the district nurse took this role, whereas in the small municipality the general practitioner and the project manager and in the urban district the health center manager and a physiotherapist were the driving force.

*Liaison*: The logbooks highlighted the importance of having an individual with social brokering skills to establish connections and create new contacts. The cross-domain nature of these networks, requires a liaison who can effectively communicate and build connections on every societal level. In the rural village it was the community support worker, whereas in the small municipality it was the project manager who executed this role.

*Change agent*: Logbooks revealed that having an individual with expertise in change management would be valuable to deal with the complexity of developing community-involved cross-domain networks. This was also mentioned by some interviewees. Change agents possess the skills to orchestrate this process. For instance, after initial difficulties to engage citizens, the small municipality appointed a project manager with knowledge of change processes. In the rural village the core group had regular access to a professional change agent for consultation.

### Competencies and expertise

*Leadership skills*: Logbooks showed that network leadership included the ability to develop a vision, see opportunities, connect with people and manage the process. Therefore, network leaders must combine various leadership and management skills. In all three networks, we observed shared leadership. This was partly due to the fact that it was volunteer work, allowing leadership tasks to be distributed more effectively.

*Determination and resilience*: From interviews and logbooks it became clear that developing cross-domain collaboration is a complex, lengthy process in which partners encounter multiple barriers. This requires *patience and perseverance* and a *learning attitude* that frames setbacks as a learning opportunity. The dynamic nature of cross-domain networks requires partners to be *flexible and creative* and *reflexive* because sustainable networks require maintenance. The rural village regularly held core group meetings to reflect on their activities, using their mission and vision statement as a guide. In the urban district, they learned during the study how to organize such meetings, which helped them to understand their strengths and weaknesses and develop shared goals.

## Discussion

This study applied a participatory action approach to provide better insights into the development of community-involved cross-domain collaborative networks and factors that facilitate or hamper their growth and sustainability. We identified novel information about the progression of networks over time, relationship-building in cross-domain networks, and key roles and competencies needed for sustainable collaboration. All three networks progressed, though one advanced only in professional collaboration, but not with citizens. Key factors identified for network development included a sense of urgency and community, collaboration mode, the importance of a change agent and the indispensability of determination and resilience.

The findings show that, both, a top-down and bottom-up approach faced the challenge of bridging the gap between formal and informal care and support systems. Also, even within the same network, the development of connections with professionals and citizens did not progress in parallel. Earlier studies on community engagement revealed that professionals may not recognize citizens as equal partners, may distrust the community’s capabilities, and may have misperceptions of the value of participatory programs [36,37]. Additional barriers for community involvement are unclarity of what community involvement entails, community’s distrust in participatory programs and cultural barriers [37,38]. Professional organizations define citizen engagement at a consultation or communication level, positioning ownership within the organizations, whereas communities define citizen engagement as active participation with ownership in the community [39]. To foster collaboration and shared ownership, previous research emphasized early citizen involvement, a safe and trusting environment and shared decision-making and governance. Other key factors for successful network development include shared goals, transparency, physical presence, informal meetings and building trust and leadership from the outset [24,25,40,41,42]. Therefore, to build trust and avoid challenges in establishing connections later on, the community and professionals from the formal care and support system should engage in a joint, transparent process of cross-domain network development from the very beginning.

Determination and resilience were identified as important factors in the non-linear, time-consuming process of community-involved cross-domain network development. Communities in our study progressed one step forward within the ESSA model. Previous studies have mentioned non-linearity and the time-consuming nature of network establishment, even among health professionals only [43,44,45,46]. Given the diversity of organizations and communities involved and the voluntary nature of citizen participation, it is reasonable to assume that fostering collaboration, building trust, and joint decision-making becomes even more complex and time-intensive in cross-domain networks [47,48,49,50]. This also supports the finding that maintaining the network requires continuous attention.

In our study we identified several key roles for sustainable collaboration in cross-domain networks. A driving force and liaison were identified earlier as part of network leadership [51]. We also identified the value of expertise in change processes or the presence of a change agent. The benefit of change agents has been emphasized in various healthcare settings [52,53,54]. In their Nine Pillars of Integrated Care, the International Foundation for Integrated Care advocates ‘collective responsibility in leading and managing transformational change and enabling individuals and the system to be their own change agents’ [55]. However, given the complexity of the process of developing a community-involved cross-domain care and support network, expertise on change processes seems indispensable. Change agents are expected to be systems thinkers, possess interpersonal, anticipatory and strategic competences, but also subject specific and normative competences, and action skills that enable them to manage this process [56]. Apart from being a consulter and researcher the change agent is expected to be a trainer [57]. This includes helping clients develop the capacity to be their own change agents, which is mentioned as one of the seven key roles of a change agent [58]. Therefore, involving a change agent is expected to benefit sustainable network development.

In line with our findings, previous studies also identified mutual understanding among partners as preconditional for sustainable cross-domain networks [59,60]. Within the medical domain, the importance of effective interprofessional and patient-provider communication for high-quality care has been emphasized in various settings [61,62,63,64]. In cross-domain networks, avoiding misunderstandings arising from profession- or citizen-specific terminologies, presents even a greater challenge [59]. Therefore, investing in mutual understanding by discussing and clarifying these differences is crucial. However, given the dynamic nature of network development and evolving network objectives, this should not be viewed as a one-time effort, but as an ongoing investment.

### Strengths and limitations

Although the ESSA-model of network development requires further validation, it served as a useful framework for the evaluation of network development in the three communities. This, combined with the reflexive character of the study, where interim results were discussed with the three networks, the advisory board and the steering committee, ensured a more valid understanding of the process of cross-domain network development. Also, the cases varied significantly in several characteristics allowing for insights into network development across different contexts. The participatory approach was experienced as positive, as researchers not only gathered data but also applied their expertise to support network development. In addition, it gave network members the opportunity to contribute to the research process.

A limitation of the study is the number of communities included, restricting external validity of the findings. However, given the diversity of the three networks, we expect our findings to contribute to the body of knowledge of community-involved cross-domain network development. Another limitation concerns the limited number of 20 interviews involving 28 respondents. Achieving theoretical saturation in such heterogeneous partnerships would require a larger number of interviews [65,66]. Nonetheless, by including all relevant stakeholders, including citizens, healthcare and welfare professionals, and policy makers, we obtained a sound overview of the factors influencing cross-domain network development. Finally, people with lived experience were involved in the execution, data interpretation, and reporting, though not in the initial design of the study. However, the application of a participatory action approach granted them significant influence over how the study evolved over time.

## Conclusion

The development of community-involved cross-domain care and support networks is a complex and time-consuming endeavor that requires patience, perseverance and a learning attitude from all partners involved. A sense of urgency or added value and a strong sense of community among community members seem to be a prerequisite. Such networks evolve through co-creation, necessitating collaboration between professionals and citizens from the start. Next to network leadership, combining diverse leadership and management skills, change agent’s expertise is recommended. Given the dynamic character of community-involved cross-domain networks, partners need to be aware that creating mutual understanding and network development is an ongoing process.
